# Bacterial Colonization of Mobile Phones: Myth or Reality

**DOI:** 10.7759/cureus.60060

**Published:** 2024-05-10

**Authors:** Sai Sravani Sure, Cunnigaiper Dhanasekaran Narayanan, Anish Kumaran N, Nithyapriya Chandramohan

**Affiliations:** 1 Department of Medicine, Sri Ramachandra Institute of Higher Education and Research, Chennai, IND; 2 Department of General Surgery, Sri Ramachandra Institute of Higher Education and Research, Chennai, IND; 3 Department of Microbiology, Sri Ramachandra Institute of Higher Education and Research, Chennai, IND

**Keywords:** meticillin-resistant staphylococcus aureus, meticillin-sensitive staphylococcus aureus, hospital acquired infections, antibiotic resistance, disinfection, mobile phones

## Abstract

Introduction

Bacteria tend to persist on mobile phones for longer durations causing hospital-acquired infections. This is primarily because mobile phones have become an extended hand to healthcare workers due to their unavoidable utilization and the lack of sanitization after use in wards.

Methods

A questionnaire was used to assess the usage and disinfection practices of mobile phones among medical students regularly attending the wards of a teaching hospital. Culture was done to assess the presence of bacteria and their resistance to antibiotics. Three sterile cotton swabs were performed for each mobile phone. If growth was present, then a culture smear was made, and the type of bacteria was assessed. Participants received subsequent education on the disinfection of phones according to standard disinfection protocol. The main objective of the study was to determine the presence of bacteria on students' mobile phones and its resistance to antibiotics.

Results

A total of 103 medical students took part in the study, which included 51 males and 52 females. It was found that all the students used their mobile phones at all times in wards and 43% of them carried their phones to washrooms. Out of all the students surveyed, only 23% of students had regularly disinfected their phones. Bacteria were present on all mobile phones sampled. Among these, 98.05% had Gram-positive bacteria, 82.52% had Gram-negative bacilli, 33.98% had Bacillaceae, and 8.73% had vancomycin-resistant enterococcus (VRE). Among participants who did not disinfect their phones, 95.89% and 97.59% had methicillin-resistant Staphylococcus aureus (MRSA) and methicillin-sensitive Staphylococcus aureus (MSSA), respectively.

Conclusion

Following standard disinfection protocols is the need of the hour to reduce hospital-acquired infections.

## Introduction

The dependence on mobile phones due to their wide range of benefits such as medical applications has made them a part of the healthcare system [1]. Mobile phones have been demonstrated to speed up communication among members of the healthcare system’s team. They are becoming essential due to their use in a variety of clinical and educational settings, such as tele-microscopy, medical imaging, and enhancing patient-doctor communication [2]. They act as fomites [3,4] and have the potential to spread bacteria among staff members, patients, and the general public [5].

A wide range of surfaces in hospitals, computer keyboards, liquid nitrogen freezers, uniforms of staff, stethoscopes, tourniquets, air, and even leaving sterile trays open for an extended period are all potential sources of the spread of pathogens that can cause hospital-acquired infections [2]. Carrying phones to toilets can also cause transmission of drug-resistant bacteria such as vancomycin-resistant enterococci (VRE). Palms and pockets act as major sources of bacterial transmission because of the moist climate, along with the optimal temperature of the human body [6]. The unintentional violation of hand hygiene protocol can be attributed to cell phones, as demonstrated by the example of a nurse taking a call while cleaning her hands as well as forgetting to wash them again prior to giving her patient intravenous antibiotics [2].

Antibiotic drug resistance is currently a widespread problem that has raised healthcare costs, increased treatment failure rates, and increased morbidity and mortality [3]. Hospital-acquired infections continue to be a reason for concern due to higher morbidity, hospital stay, and mortality. In addition to direct transmission, there is a possibility of indirect transmission through medical personnel's hands and other medical equipment becoming contaminated [2].

In addition to showing an increased rate of bacterial contamination, the utilization of mobile phones by the workers of healthcare in the ICU and the operating room also revealed a higher rate of nosocomial pathogen contamination. It was also reported that microbes transferred from hand to mouth during casual activities after handling contaminated food [7].

Mobile phone covers and cracked screens raised the risk of contamination, while cleaning the phone reduced it. Participants' cell phones were also substantially less contaminated when they reported having cleaned them with an alcohol swab in the last 24 hours [2]. Studies conducted in developing countries have revealed a higher incidence of mobile phone contamination than those conducted in developed countries [4,8]. They have the potential to act as reservoirs for community-associated (CA)-methicillin-resistant Staphylococcus aureus (MRSA), healthcare-associated (HA)-MRSA, and methicillin-sensitive Staphylococcus aureus (MSSA) strains [8]. This study determined to assess the presence of bacteria and disinfection practices of mobile phones followed by the medical students regularly attending the wards.

## Materials and methods

Study design

A cross-sectional study was performed among 103 medical students (including 51 males and 52 females) within the laboratory of the Department of Microbiology, Sri Ramachandra Medical College, from July 2023 to December 2023. 

A sample size of 103 was obtained by taking p=0.04 from the article:

“The Bacterial colonization of healthcare workers’ mobile phones in the ICU and effectiveness of sanitization” [1].

Data and sample collection

A questionnaire was made to obtain demographic details (name, age, sex) and to evaluate the use of mobile phones and disinfection practices among study participants.

The whole procedure of sample collection was carried out in clean and aseptic conditions. The instruments used for the sample and data collection procedure were sterile cotton swabs, test tubes, petri plates, incubator, inoculation loop, Whatman filter paper, glass slides, nutrient agar, Mac Conkey agar, Muller-Hinton agar, thioglycolate broth, urease agar, mannitol motility medium, Penicillin and Pimaricin (PPA) agar, citrate agar, triple sugar iron (TSI) agar, bile-esculin agar, hydrogen peroxide, Kovac reagent, indole reagent, cefoxitin, cefotaxime, clavulanic acid, imipenem, and vancomycin.

Using sterile cotton swabs, three swabs were performed for each mobile phone. Front surface, back surface, and side surfaces of mobile phones. Followed by which, the swab was inoculated into thioglycolate broth and sub-cultured on blood MacConkey agar. Then, it was incubated for 48 hours. If growth was present, then a culture smear was made. The biochemical reactions used for Gram-positive cocci are catalase, urease, mannitol motility medium, tube coagulase test, and bile-esculin test. Drug sensitivity was tested for MRSA and VRE using cefoxitin and vancomycin. The biochemical reactions used for Gram-negative bacilli are catalase, oxidase, indole, TSI test, urease, citrate, mannitol motility medium, and PPA test. Drug testing was done for extended-spectrum beta-lactamase (ESBL) and ceftriaxone using antibiotic discs cefotaxime, clavulanic acid, and imipenem.

Participants received subsequent education on the importance of disinfection of phones according to standard disinfection protocol.

Methodology of the study

**Figure 1:**
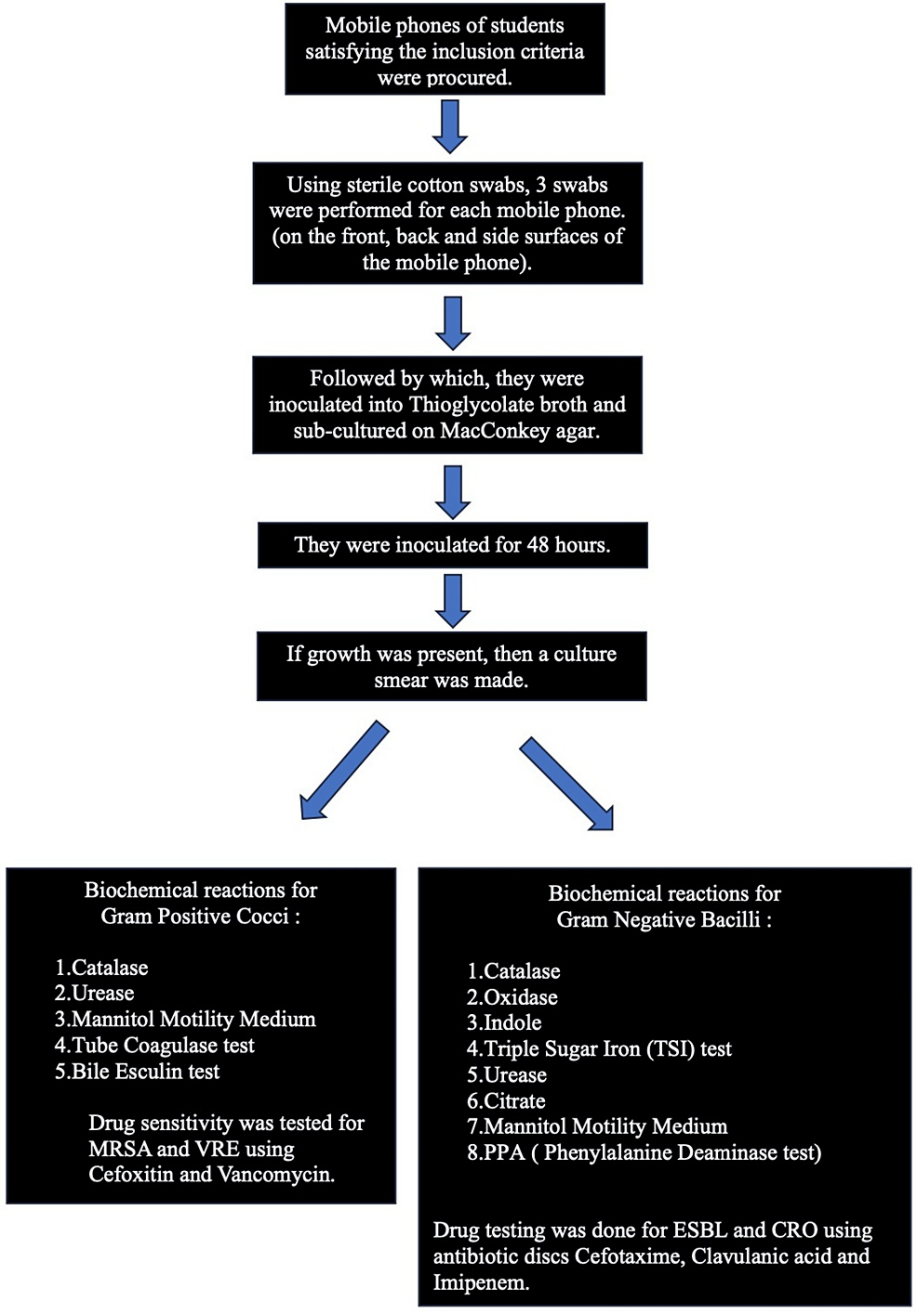
Flowchart depicting the methodology of the study

Figure 1 presents the methodology of the study.

Data analysis

The data analysis was done using Statistical Product and Service Solutions (SPSS, version 16.0; IBM SPSS Statistics for Windows, Armonk, NY). Descriptive statistics and proportions were used. Mean standard deviation and their differences were used.

Ethics approval

Institutional Ethics Committee (Sri Ramachandra Institute of Higher Education and Research) approval was obtained (IEC approval number: CSP /23/MAY/128/404).

Informed consent has been attained from all the participants along with the students satisfying the inclusion criteria who participated in the study; medical students attending the hospital wards of Sri Ramachandra Medical College and who have used their mobile phones for more than six months have been involved in the research. Medical students not attending the wards were not included in the study.

## Results

Demographic details of the participants

One hundred and three medical students participated in this study (including 51 males and 52 females). The mean age of the participants was found to be 21 years (SD: 2.127). It was found that all the participants had spent a minimum of three hours with the patients in the wards.

Use of mobile phones by the participants

All the students had carried and used their phones at all times in wards and operating theatres for various reasons such as referring to online textbooks, taking notes, calculating dosages of medications, etc. Out of all the medical students attending the wards surveyed, 43% of them had carried their mobile phones to the toilets.

Disinfection practices and its awareness among participants

It was found that not all participants found it essential to disinfect their phones. Only 56% of all the participants surveyed found it essential to disinfect phones regularly, and only 32% of them were aware of proper methods of disinfection of mobile phones. Even among the students who were aware of proper disinfection methods, only 23% of them had regularly disinfected their phones. Among the students who were aware of standard disinfection measures, the source of information was lectures attended at medical school followed by posters and articles on standard disinfection protocol. The responses obtained on reasons for cleaning mobile phones included preventing transmission of infections to patients, preventing antibiotic drug resistance among patients, preventing diseases among participants and their family members, and keeping their phones clean. The reasons for not cleaning their phones included a lack of awareness of the importance of disinfection of phones, not having sanitizer, having no time to follow disinfection protocol, and fear of mobile phones getting damaged.

Bacterial contamination of mobile phones

All mobile phones had bacteria present on their surface and cases. A total of 101 phones had Gram-positive bacteria, 18 had only Gram-positive bacteria, 94 had Gram-positive cocci in clusters, 28 had Gram-positive cocci in tetrads, and 26 of them had a combination of both clusters and tetrads. Bacillaceae were found on 35 phones. Additionally, 85 phones had Gram-negative bacilli, two phones had only Gram-negative bacilli, and a total of 83 phones had a combination of both Gram-positive and Gram-negative bacteria. Further, a total of 70.87% and 80.58% of mobile phones had MRSA and MSSA, respectively. Among students who did not disinfect their phones regularly, MRSA and MSSA were found to be 95.89% and 97.59%, respectively. Nine phones had VRE present on them.

**Figure 2:**
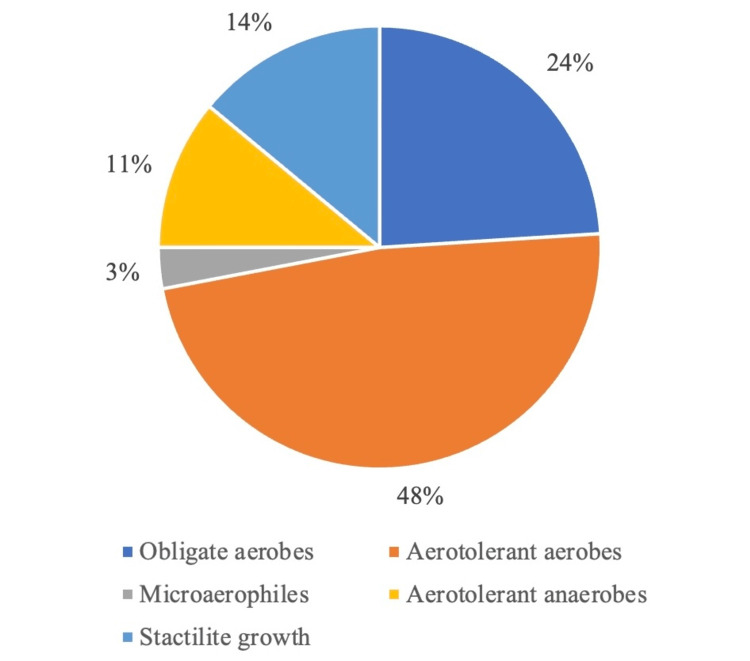
Types of bacteria found on mobile phones

From the results obtained after sampling the mobile phones, it was found that most of the bacteria were the ones that colonize and tolerate aerobic environments. Among all the types of bacteria, aerotolerant aerobes (48%) and microaerophiles (3%) had the highest and lowest prevalence, respectively (Figure 2).

Among all the bacteria isolated, MSSA and MRSA were predominantly and commonly isolated from the majority of mobile phones, i.e., 80.58% and 70.87%, respectively. Among Gram-negative bacteria, pseudomonas has the highest prevalence (58.25%) (Table 1).

**Table 1:** Pathogenic bacteria isolated from mobile phones

Organism isolated	Number	Percentage
Gram-positive bacteria		
Meticillin-resistant Staphylococcus aureus	73	70.87
Meticillin-sensitive Staphylococcus aureus	83	80.58
Streptococcus	1	0.97
Presumptively Streptococcus species	5	4.85
Enterococcus	30	29.12
Staphylococcus	87	84.46
Micrococcus	30	29.12
Gram-negative bacteria		
Pseudomonas	60	58.25
Non-fermenting Gram-negative bacilli	56	54.36
Fermenting Gram-negative bacilli	7	6.79

The presence of MRSA (95.89%) and MSSA (97.59%) was higher and significant among the students who did not disinfect their phones regularly when compared to the students who disinfected their phones regularly (Table 2).

**Table 2:** Relationship between the prevalence of meticillin-resistant Staphylococcus aureus and meticillin-sensitive Staphylococcus aureus on mobile phones and disinfection practices followed by medical students

Disinfection practice	Number of phones that had the presence of MRSA	Percentage	Number of phones that had the presence of MSSA	Percentage
Students who regularly disinfected their phones	3	4.11	2	2.41
Students who did not regularly disinfect their phones	70	95.89	81	97.59

**Figure 3:**
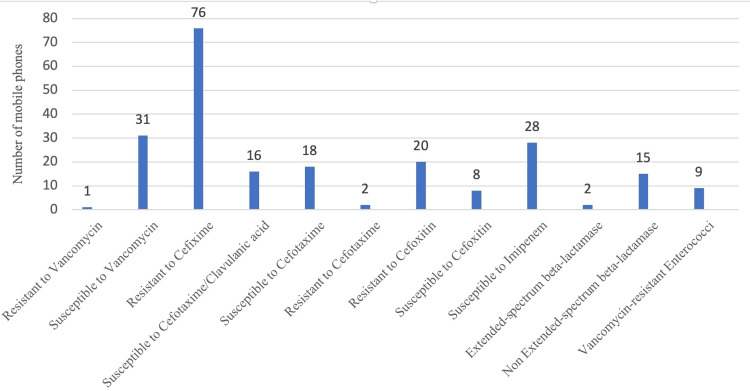
Antibiotic susceptibility of the bacteria present on mobile phones

The majority of the mobile phones (i.e., 76) among sampled were resistant to cefixime antibiotic, and one mobile phone was resistant to vancomycin. Nine mobile phones had vancomycin-resistant Enterococci on them (Figure 3).

## Discussion

The study results showed that bacteria were invariably present on all the mobile phones sampled among medical students. MRSA and MSSA were isolated from the majority of phones, and nine mobile phones had VRE. Among the students surveyed, every participant had used a mobile phone in the ward, and 43% of them reported using their mobile phone in washrooms.

Multiple research done among healthcare workers demonstrated that their mobile phones are contaminated with bacteria. According to a study conducted among healthcare workers at a teaching hospital in Lusaka, 79% of the mobile phones surveyed had bacteria on them. The presence of bacteria on mobile phones was found to be from 70% to 100% among healthcare workers in different studies done in various countries [9]. However, no study has reported 100% contamination of mobile phones surveyed exclusively among medical students attending the wards regularly.

In a study done among 183 healthcare workers at a hospital in Turkey, the prevalence of MRSA was found to be 9.5% of mobile phones sampled. Further, a study done at a hospital in Hiroshima, Japan, reported that 10% of the mobile phones tested had the presence of MRSA [10]. Our study was done exclusively among medical students attending the wards regularly and has revealed a very high percentage of contamination of mobile phones with MRSA (70.87%) and MSSA (80.58%). Among the students who did not disinfect their phones regularly, MRSA and MSSA were found to be 95.89% and 97.59%, respectively. 

The Centers for Disease Control and Prevention and professional societies have acknowledged that cleaning practices are frequently not up to par. As a result, a system for tracking compliance with suggested cleaning practices is required to guarantee that surfaces in patient rooms are consistently cleaned and disinfected [2,4,11].

Many factors influence the disinfectant's effectiveness, such as the item's previous cleaning history, the presence of organic as well as inorganic substances, the kind and degree of microbial contamination, the disinfectant's concentration as well as the time of contact, the item's physical characteristics, pH, biofilm, and surrounding temperature. The disinfectant can be applied with a wet cloth, wiped, or sprayed on [11].

Healthcare workers must be informed about the frequency and method of cleaning, the concentration of products that have to be used, and the contact time of cleaning. Mobile phone usage in hospitals should be avoided. They must be cleaned frequently using wipes containing antiseptics, such as 70% isopropyl alcohol and 5% chlorhexidine, and users should practice good hand hygiene both before and after using their phones. Disinfection of mobile phones is a crucial part of the infection control strategies for healthcare workers and the environment [5]. The areas of work that require the highest standards of hygiene are hospital operating rooms (OR) and intensive care units (ICU) [5,12,13]. Carrying mobile phones to toilets should be strictly avoided [14]. Further, the duration of viability of bacteria on mobile phones acts as a major risk factor for its transmission among various departments, thereby making disinfection essential [15]. The proportion of bacteria resistant to stronger antibiotics is on the rise; therefore, patients afflicted with nosocomial infections must be treated with combinations of stronger antibiotics. Every time a phone call is made, the hands come into contact with highly contaminated human body parts, such as the mouth, nose, and ears [5].

Overall, the goal of our study is to contribute knowledge regarding the fact that mobile phones serve as fomites and result in the spread of potentially life-threatening bacteria such as MRSA, MSSA, and VRE. This study underlines the need for stringent measures and standardized disinfection protocol to be practiced by all healthcare workers, including medical students, making mobile phone disinfection a procedure that must be adhered to strictly.

Limitations of the study

This is a single-centre study that was restricted to medical students attending the wards regularly. This study identified the presence of bacteria on participants' mobile phones but did not compare it to the presence of bacteria after providing health education, thereby not evaluating the efficacy of disinfection practices. Future studies can be done by carrying out pre- and post-sampling of mobile phones to assess the effect of standard disinfection practices among healthcare workers' mobile phones.

## Conclusions

The versatile use of mobile phones at the hospital makes it almost impossible to avoid them. Medical students play a major role in the bacteria transmission in a hospital setting due to their continuous interaction with the patients. Many patients admitted to the wards would be immunocompromised or resistant to a few or multiple drugs. This puts them at a higher risk of hospital-acquired infections, thereby increasing their hospital stay and morbidity. Therefore, stringent standardized disinfection protocol has to be followed by all healthcare workers to decrease the risk of MRSA, MSSA, and multidrug-resistant bacteria transmission. Further, all the healthcare workers, especially medical students attending the wards, have to be made aware and taught about the standard disinfection methods, and their adherence to the protocol has to be monitored regularly.
